# Providers’ perspectives on implementing resilience coaching for healthcare workers during the COVID-19 pandemic

**DOI:** 10.1186/s12913-022-08131-x

**Published:** 2022-06-14

**Authors:** Benjamin Rosen, Mary Preisman, Heather Read, Deanna Chaukos, Rebecca A. Greenberg, Lianne Jeffs, Robert Maunder, Lesley Wiesenfeld

**Affiliations:** 1 Sinai Health, Toronto, Canada; 2 Department of Psychiatry, University of Toronto, Toronto, Canada; 3 Lunenfeld-Tanenbaum Research Institute, Sinai Health, Toronto, Canada; 4 Department of Paediatrics, University of Toronto, Toronto, Canada

**Keywords:** Resilience, Coaching, Burnout, Healthcare, Support

## Abstract

**Background:**

The COVID-19 pandemic severely exacerbated workplace stress for healthcare workers (HCWs) worldwide. The pandemic also magnified the need for mechanisms to support the psychological wellbeing of HCWs. This study is a qualitative inquiry into the implementation of a HCW support program called Resilience Coaching at a general hospital. Resilience Coaching was delivered by an interdisciplinary team, including: psychiatrists, mental health nurses allied health and a senior bioethicist. The study focuses specifically on the experiences of those who provided the intervention.

**Methods:**

Resilience Coaching was implemented at, an academic hospital in Toronto, Canada in April 2020 and is ongoing. As part of a larger qualitative evaluation, 13 Resilience Coaches were interviewed about their experiences providing psychosocial support to colleagues. Interviews were recorded, transcribed, and analyzed for themes by the research team. Interviews were conducted between February and June 2021.

**Results:**

Coaches were motivated by opportunities to support colleagues and contribute to the overall health system response to COVID-19. Challenges included finding time within busy work schedules, balancing role tensions and working while experiencing burnout.

**Conclusions:**

Hospital-based mental health professionals are well-positioned to support colleagues’ wellness during acute crises and can find this work meaningful, but note important challenges to the role. Paired-coaches and peer support among the coaching group may mitigate some of these challenges. Perspectives from those providing support to HCWs are an important consideration in developing support programs that leverage internal teams.

## Background

The COVID-19 pandemic exacerbated pre-existing workplace stresses for healthcare workers (HCWs) worldwide. Principles for supporting HCWs’ psychological wellbeing are well described [1–3], and the need has been clearly articulated [4, 5]. Existing literature on HCW support programs during COVID-19 largely focuses on program description and its impact on participants [6–12]. Perspectives on the experience of providing support during COVID-19 are an important dimension of workplace resilience that is less often reported [13, 14]. This paper explores the perspectives of mental health clinicians who delivered a specific support program called Resilience Coaching [14, 15]. Sharing these perspectives can facilitate deeper understanding of development, implementation and sustainability of HCW support programs during a public health crisis such as a global pandemic. Lessons learned may help other institutions to develop programs of their own.

### Principles of staff support programs

Research from previous outbreaks of infectious disease, including SARS and H1N1 influenza, indicates that pandemics are associated with increased rates of HCW burnout [16–22]. As of Spring 2021 (one year into the COVID-19 pandemic), the rate of severe burnout amongst healthcare professionals as a group was estimated to be over 60% [23]. The Registered Nurses Association of [the region] reported that in the summer of 2021, nurses are leaving the profession in much higher rates than usual, because of pandemic related burnout, leading to critical staffing shortages that threaten health care operations [24].

At the beginning of the pandemic, the need to urgently develop psychological support programs for HCW was apparent [1–3]. HCW Burnout mitigation recommendations include: rotating staff between high and low stress areas, establishing visible leadership, transparent and clear communication of policies, and allowing staff time and space within the workday to voluntarily decompress with the assistance of trained professionals [1, 2]. Worldwide, a range of HCW support programs have been developed, including: mobile phone applications, online courses, establishment of research centres on HCW wellness, and in-person on-the-ground support that includes peer support, onsite resting rooms for HCWs, and the presence of psychologically trained staff at team meetings [6–12].

Program goals focus on increasing HCW resilience in the face of stress. In the context of a pandemic, it is important to note that resiliency interventions and supports alone cannot and should not replace general hospital disaster readiness and infection prevention and control measures; instead, we advocate that they are part of an overall approach to hospital pandemic readiness.

### Supporters’ experiences of delivering support

Research about provider’s experiences of delivering staff support programs is limited. One qualitative study of mental health providers supporting frontline HCWs during COVID-19 in the United Kingdom found that providers were motivated to offer support, but that they also experienced concerns including: uncertainty about their abilities, a blurring of clinical and collegial boundaries, and vicarious trauma and isolation [13, 14]. Because Resilience Coaching is delivered by mental health professionals, it is relevant that research about the experiences of mental healthcare providers working during the pandemic reveals similar trends in the areas of motivation, uncertainty about abilities and isolation [2, 25–27]. It has also been suggested that people providing support in crisis situations are at risk of overidentifying with those that they support, so connection between like-supporters is critical to help providers maintain appropriate professional boundaries and practices [28]. Tending to the wellbeing of mental health providers supports the entire healthcare system [29]. Greater understandings of the experience of providing mental health support during the pandemic is an important consideration.

## Methods

### Setting and aim

Resilience Coaching took place at a general hospital and academic medical centre in Toronto, Canada.

In this paper, we aim to explore the experiences of mental health professionals who worked as Resilience Coaches delivering the Resilience Coaching staff support program to their direct colleagues at the same organization, from April 2020 to June 2021. We describe their motivations and barriers to delivering the intervention. By describing experiences of Resilience Coaches, this research may assist other healthcare organizations in understanding what is required to implement similar programs, and inform future pandemic preparation [30].

### Resilience coaching – intervention description and design

Resilience Coaching uses principles of consultation-liaison psychiatry and psychotherapy to provide collegial support in a timely manner and focuses on the development and maintenance of supportive relationships. It was developed by a team of mental health professionals in response to an urgent call for support for HCWs [1–3] and was informed by prior work at [hospital] where the department was involved in supporting HCWs during previous pandemics, SARS in 2003 and H1N1 in 2009 [16–22]. The program is funded by COVID-19 specific funding, which was created in early 2020.

Resilience Coaches are mental health professionals, also working on the frontlines of the same hospital as the coaching intervention, who bring skills rooted in psychotherapy, psychological first aid and stress management to clinical settings such as huddles, staff meetings and ward rounds to facilitate support [14, 15, 31, 32]. Key components of this coaching intervention are *familiarity* (support is more easily accessed and accepted if it takes place in the context of a consistent and trusting  relationship) and *flexibility* (support should be offered on a time scale and in a location that meets staff needs, and via multiple methods of delivery). Resilience Coaching draws on principles of consultation-liaison psychiatry, including the importance of the psychiatrist being embedded or affiliated within medical and surgical teams to provide patient care; here, the focus of support is the clinical team, rather than patients [15]. Coaches participate in a weekly peer support group for themselves with the coaching group to refine coaching practice and interventions and assist in maintaining their own wellness.

When possible, in keeping with a consultation/liaison frame, coaches are encouraged to work with hospital units with which they had pre-existing relationships. When this is not possible, Resilience Coaches work to establish a consistent presence with the team, to foster trust and consistency. Operationally, Resilience Coaching is flexibly tailored to the structure and functioning of the clinical teams they support. Prior to meeting, coaches liaise with the unit manager to inquire about the staffing setup, schedules, and perceived needs of the unit. In practice, some units expressed a preference to incorporate coaching into existing team meetings whereas others preferred to establish new meetings devoted to coaching and staff support. Typically, during the initial meeting, coaches solicit feedback from the group on whether additional sessions are desired as well as the optimal setup, timing and frequency of meeting. Coaches offer meetings for as long as they are desired by the unit and as long as they have capacity to do so, in the context of competing clinical demands.

Coaching tends to occur in small groups of 5–10 staff at a time. There is no cap placed on group numbers; larger groups are rare, but welcome. Infrequently, coaches meet with individual staff to determine the best course of support and facilitate referrals when indicated. In terms of content, Resilience Coaches provide psychoeducation about normal stress and coping, burnout and when to seek further help. Coaches offer to facilitate strategies for stress reduction such as mindfulness or meditation, or run group activities to promote team cohesiveness. Initially, Resilience Coaching was offered to staff working in clinical areas where impacts of pandemic policies were expected to be most acutely felt, including the Emergency Department and Intensive Care Unit. Over time, Resilience Coaches received requests for support from a range of areas throughout the organization, both clinical (such as pharmacy, nutrition, sports medicine, and others), and non-clinical (such as administrative and foundation, and entry screeners). The frequency of coaching sessions fluctuate based on the group’s bandwidth to offer support, as well as staff need as assessed by the Resilience Coaches or expressed by staff or managers.

### Qualitative inquiry – Data collection and analysis

This qualitative research study of Resilience Coaching began in the fall of 2020. Research was led by two coaches (BR, MP), with support from a research assistant with a background in education and training in ethnography and oral history (HR). A senior psychiatrist and coach (RM), a senior health services researcher (LJ), and two additional coaches (DC, RG) participated in analysis of the interviews. [HR] interviewed Resilience Coaches who had been working for more than 3 months in this capacity. Participants were invited to be interviewed via emails distributed by departmental heads, and self-identified to the interviewer. Participants provided written informed consent and the study was approved by the [hospital] Research Ethics Board.

Hour-long semi-structured interviews were conducted by [HR] using the hospital’s virtual meeting platform. Interviewees were asked about their demographics, followed by their experience of living and working during the pandemic, and what it was like to provide coaching. Interviews were professionally transcribed. The team conducted a thematic content analysis, identifying themes and subthemes within the interviews [33–35]. [HR] conducted initial analysis on a subgroup of transcripts to inductively establish the main themes, and [initials] completed the coding on the remaining data. The team used NViVo to track the themes across the dataset, and refined thematic categories through regular group discussion with the wider research team [initials]. To further inform analysis the emerging themes of interviews were cross checked against notes from the peer support meetings, which contain themes discussed among the group of Resilience Coaches [35].

This paper focuses on the experiences of the Resilience Coaches, both delivering Resilience Coaching and more generally during the pandemic^1^; we have a specific focus on their motivations and challenges in delivering the intervention, as well techniques used to run sessions for staff.

## Results

### Participant demographics

Out of 15 total coaches, 13 participated in interviews: 9 psychiatrists (70%), 2 mental health nurses and 2 non-clinical hospital staff (15% each). Eight coaches self-identified as female (62%) and 5 self-identified as male (38%). 

### Coaching frequency and attendance

The frequency of coaching sessions varied considerably depending on the clinical area and perceived level of stress amongst staff, which often correlated to spikes in hospital or community case counts. As an example of frequency, from January to March 2021, which was one of the most acute phases of the pandemic in this region, 12 coaches reported running 168 sessions across the two hospital sites.

Staff attendance at coaching sessions varied considerably, and seemed to depend on a variety of factors, including: the format of delivery, departmental culture, subject of the session, and status of COVID-19 infections in the area. For some units, virtual coaching was more popular, and for others, in person was preferred. Moreover, preferences and attendance shifted throughout the pandemic. Units with established large meetings often offered coaching sessions as part of these meetings, which were often well attended; however, during times of increased infection counts, virtual coaching sessions often became more popular, because they could provide more opportunities for quiet reflection. These factors are discussed in more detail throughout via examples from the participating coaches.

Most frequently, staff attendance was reported in the range of 1–8 people at a session.

### Resilience coach interview themes

Three main themes emerged from the analysis: A) Motivations and rewards, B) Challenges, and C) Coaching strategies and techniques.

#### A) Motivations and rewards

Coaches described their experience as rewarding – providing satisfaction, connection, and a meaning. They frequently reported feeling honoured to support colleagues during a difficult time. One said it was a privilege for people to “open up and share their real accounts…and let me in in that way.” (C1). For another, providing support “feels helpful and useful.” (C2). Another reported it was “beautiful to bear witness to the teamwork and to the resilience of the units” and that “there aren’t enough pots and pans in the world to recognize what nurses are doing” during COVID-19 (C3).^2^

Delivering Resilience Coaching also provided some coaches with a deeper understanding of life within their hospital. One said Resilience Coaching helped her “feel connected to everybody in a different way,” that “there are a group of people that get it,” in contrast to people outside whose professional lives had not been as severely affected in the same way (C4). Another noted that providing Resilience Coaching to the unit they supported, where they also have professional connections, improved their understanding of challenges experienced by that team. They noted this improved understanding was proving helpful in their regular role on the team, where they worked with the team providing the mental health component of “collaborative care” for their patients (C5). Another coach commented that delivering Resilience Coaching made them more aware of “power and privilege” in the hospital, especially related to discrepancies between nurses and physicians (C3); several others expressed this idea as well. For these coaches, it was personally and professionally meaningful to have a deeper understanding of the realities of working lives of their colleagues, and renewed their initial desire to help.

#### B) Challenges

##### i. Role tensions

Coaches described challenges in their role that included uncertainty about their roles and questions about the scope of their expanded role within their organization. One coach noted feeling uncertain about delivering sessions that were not explicitly requested by staff: “No it’s not easy and natural…I don’t offer my service unless people ask me [laughs]. So, I feel like I don’t want to force this on people who are so busy already” (C6). Another noted that perceived power imbalances influence sessions; they felt an “odd tension” because they didn’t “want to be the doctor in the room.“ (C2). For this coach, Resilience Coaching became challenging over time:

I feel increasingly uncertain about the sessions. I thought at the beginning that our role was really…a responsive role and that…I just had to show up and help deal. And that's the role I can do, that's OK. With time going on, there's less fires, but the temperature is still hot. And I keep wondering [about what we should do]…(C7).

Another key challenge was a role tension about maintaining boundaries between clinical care and Resilience Coaching. One coach articulated the difference: “operationally, clinical care requires identifying a patient, opening a chart, keeping notes. You bill for that, and you have a regulatory responsibility to your college…So the nuts and bolts of that are is really clear…[But] why we choose that for one person…and [not] for someone else…is less clear” (C8). Another noted they experienced a “blurring of…what it means to provide support” and felt a need to “constantly [be] evaluating…at what point does this conversation…need to become clinical care?” when working with staff (C3). Another coach reflected on the fact that with one staff member, they had not defined their role as distinct from clinical care. The coach described writing a letter advocating for the staff member which led them to personally question “where does the role [of the coach] actually end?” (C9).

Another tension was a lack of clarity about whether they were perceived by staff as colleagues providing support or as representatives of the hospital itself. One coach used a wartime metaphor to describe this: “I always wonder…is my role as a coach to make people…good soldiers, so that they carry on the mission of the hospital?” (C5). Another observed this tension arises when the organization’s requests and what staff are able to do are not aligned. The coach described feeling “in the middle…to figure out…is there a role for us to do something, or do we just focus on the…coaching aspect of things?” (C10).

##### ii. Logistics

Coaches noted several logistical challenges in the delivery of Resilience Coaching, often related to scheduling or finding a space for sessions. In one situation, a coach noted one group had a private space, but it was “also a medication room, and there's pharmacy delivery that's always in the middle of [the meeting].” For their other group, there was no private room, so they used the staff lounge. The coach reflected “usually, we have to kick people out of the lounge,” which felt uncomfortable (C4). Night shift workers and physicians, in particular, were noted as challenging to schedule. Some coaches also described a limitation on being able to arrange sessions to meet staff needs, due to constraints of their own clinical work. Additionally, many coaches reported setting meetings that were unattended or rejected. Rejection happened during summer months, and during COVID-19 outbreaks. These experiences raised for some a question about how much their time to offer, when, as one coach noted “every hour we spend with a group coaching is an hour we could spend doing a patient group” (C11).

Most coaches were interviewed approximately one year into the COVID-19 pandemic, and described a range of personal life stressors that they too experienced during that time. As HCWs themselves, they were not immune to the pressures and stresses experienced by those that they supported as Resilience Coaches.

##### iii. Coping with burnout

Most coaches were interviewed approximately one year into the COVID-19 pandemic, and described a range of personal life stressors that they too experienced during that time. As HCWs themselves, they were not immune to the pressures and stresses experienced by those that they supported as Resilience Coaches.

Coaches recounted experiencing considerable fear and anxiety in the early pandemic, but at the time of interview, it was more common for them to describe coping with those feelings. Exercise and cooking were common coping strategies described, as well as spending time with loved ones, and taking time alone to decompress when needed. While generally appreciative of their privilege, coaches also noted difficulties, such as using alcohol more frequently, and struggling to maintain a boundary between work and home life.

A common source of strain among coaches related to the impact of the pandemic on family life. Several Resilience Coaches described stresses of pandemic parenting, as in this individual who noted “it just feels like I've let my kids down…I just keep reminding myself that in March whenever they announced the first shutdown, I was like how are we going to survive…? And I just keep reminding myself that at that point three weeks felt impossible and now it's been much longer.” (C4). At the other end of the life cycle, some Resilience Coaches had elderly parents in their care, as in this example:

I talk to my parents every day which is both, you know, a plus and a negative because I worry about them a lot. They’re quite elderly… they’re fortunately not in long-term care, they’re in their own apartment but it feels scary for them. And my father is having declining health and so that all feels difficult. But I would say talking to them is more helpful in remaining resilient that not. (C5)

This coach noted that while they felt privileged and grateful for the experience of their immediate family, the pandemic was still a strain, resulting in feelings of burnout affecting their clinical work: “I think I just feel tired. I think my tolerance for patients is really low. So, I feel like that’s where probably the biggest impact has been which is that…I just don’t have the capacity to deal with really needy patients…like it gets under my skin and irritates me.” (C2).

Some coaches explicitly highlighted the challenge of providing support while experiencing burnout. One described, “there is some pressure to be well, because we are the ambassadors of resilience…And that’s tricky, too, right?…we can’t always give an answer about what we’re personally doing around resilience, because we’re not always doing it” (C10). Another stated they found leading coaching sessions sometimes magnified their personal difficulties: “I would also say that my own experience of the pandemic…makes it very hard to be a coach to other people. Because most of the time I'm in a very negative place in terms of what's going on for me personally. And when I'm in the session I just actually feel like even more terrible for what other people are going through.” (C4).

#### C) Coaching strategies and techniques

##### 1. Relationship building, establishing trust and validation

Relationship building was a commonly described strategy used by Resilience Coaches in running sessions. This coach describes creating a new relationship with a team: “I often didn't know how to support them…I mostly just tried to be visible. I wanted people to know that they weren't going to go through this alone” (C11). This Resilience Coach used humour: “And the telling a joke thing? I do that with my patients too, but I don't do it as much. Because I think the coaching thing needs to feel a little bit more contained for people. And I also feel like…they need to know this is a peer thing” (C8). Other relationship building tools described include self-disclosure, and helping the team where possible, even stepping outside of normal roles.

Coaches described being attentive to staff emotions as key to their practice, often to build trust. One described validation as a form of education:

…[I coach] to help people realize that negative emotions, negative reactions, are not necessarily a sign of failure, shortcoming, or lack of professionalism, but rather an occupational hazard of working…in healthcare at any time, [and] especially during COVID times. So, educating people to realize that there is an occupational hazard to working during COVID…it will affect you as a human being, and what you do with that feeling will help you be resilient or not…if we can talk about, and we can talk about why it’s OK to feel like that…it seems to help people. (C1).

For this coach, listening and validating were essential as well: “…a lot of the time it’s just about kind of asking a different question or listening in a different way, how can you listen in a different way that isn’t all about finding solutions, but it’s just hearing people and where they’re at.” (C12). This coach described that the practice can be “a lot of…listening, validating, reframing sometimes, where there’s room to think of things differently.” (C10).

##### ii. Psychotherapeutic techniques

Resilience Coaching is a form of collegial support, distinct from clinical care. However, coaches draw on principles of psychotherapy and psychological first aid in doing the work (1). This coach noted that: “it’s clear that [Resilience Coaching has] been informed by my psychotherapy…When it’s more on an individual level, it’s not the resilience building, it’s sort of – there’s a lot of listening, there’s a lot of…empathizing with what’s going on, and there is some intervention that goes beyond coaching, I think.” (C9).

Some coaches described specific principles derived from psychotherapeutic modalities (such as cognitive behavioural therapy (CBT) or dialectical behavioural therapy (DBT)) were useful to incorporate. This coach illustrates how this can be applied in coaching: “One of my favourite types [of therapy]…is dialectical behavioural therapy, which is the idea that two opposing things can exist at the same time. So, we spend a lot of time talking about…for example, I’m so burnt out and I’m so tired, and my job is important to me and this role has great meaning…” (C10).

Other coaches noted group therapy skills were ones they commonly used in conducting Resilience Coaching sessions. This coach was self-deprecating about their group management: “I actually have to call upon all of my meager group psychotherapy skills to try and contain…[some staff] so that the others can function as a group…” (C8). Another described the group management more broadly:

I would say that there's a lot of generalisation, a lot of normalisation, a lot of intra-group learning. Somebody raises a problem, anybody else faced that, anybody else got an idea how we can manage that? And a lot of, in a sense, encouraging the group to manage itself as opposed to offering specific interventions. That would be the stuff that feels like its psychiatric, feels like group therapy in that way…[but] they don't want therapy. They…see us as colleagues, not as therapists. (C2)

##### iii. Paired coaching

Many coaches reflected positively on the experience of delivering Resilience Coaching with a partner. This coach noted being paired made it easier to begin Resilience Coaching: “I was paired with [name]… everything he says is smart and inspiring...Since that time, after the first meeting he’s no longer doing it, but the first meeting was just made easier…” (C5). One coach noted working in pairs facilitated self-reflection: “Coaching in pairs…allows for the coach to have someone who they can bounce ideas off of and ask, how did that land when I tried that exercise with the group? Or what did you think of this.” (C3). This coach noted a possible value in working in pairs may lie in the enhanced relatability of the coaches: “there’s also a clear difference between, like, who will reach out to me versus who will reach out to my colleague, and I think that’s because we are relatable to different groups of people.” (C7). Another noted that pairs-based coaching allows for logistical flexibility “to share days when one of us needs to be away or when there’s other competing demands,” as well as echoing that having two facilitators is helpful because “we pitch things at two different levels and I think that appeals to different demographics.” (C10)

## Discussion

The COVID-19 pandemic has magnified the vulnerability of human resources in healthcare and exposed the need for a more robust approach to supporting HCWs psychological wellbeing in usual times, in crises, and in recovery. Designing the right support for the right setting is a complex endeavour and should incorporate perspectives and lessons learned from past experience. This paper focuses on describing providers’ experience of offering a support program, which are not often a focus of research [13, 14]. Given that, akin to other similar models, providers in the Resilience Coaching model were also healthcare workers, understanding their experience and the impact of these dual roles is an important sustainability and staff resilience consideration.

The results of this qualitative inquiry into the experiences of Resilience Coaching reveals coaches found the work rewarding and valuable. The embedded, relationship-based model [15] offers a framework for mental health professionals to contribute to a pandemic response using their specific skillset, and opportunities for interpersonal connection and mastery, which adds meaning to the work. The model used in this study is similar to one proposed in literature, known as ‘anchored personalization.’ DiBenigo and Kerrissey describe this as a model drawn from the US army, where mental health support providers are assigned to specific frontline units where they develop relationships with those in need. These relationships allow for the provision of immediate, on the ground support, and help break down stigma associated with seeking mental health support (the personalization). The providers are also part of a peer group of other providers, where they discuss shared issues (the anchor) [28, 36]. The duality of this structure allows for adaptive support, which remains focused and grounded by the peer discussions. Findings from our study, and ones that use similar structures of embedded, regular support, found that this model was generally well received by both givers and receivers of support [37–41].

Though this model has been found to be generally well received, Resilience Coaching is also associated with challenges for providers, which ranged from balancing competing role tensions and uncertainty about abilities, to coordinating logistics in a busy hospital environment or offering support while managing one’s own burnout.

Role tension related to whether the Resilience Coaches were perceived to be agents of the hospital, or colleagues reaching out peer-to-peer, was a common issue for some coaches early on in their delivery of Resilience Coaching. Interestingly, it has been established that when staff perceive that the organization is providing support, they experience less burnout; so, while it was a conflicting status for some coaches, the perception that they were agents of the organization was likely beneficial for recipients [40]. In future versions of this program, or others like it, we recommend emphasizing this known benefit, as well explicitly addressing this role tension in peer support meetings. Left in an unclear state, or without a way to process this tension, it may progress to becoming a goal conflict for the group or an identity conflict for the Resilience Coach (or other staff supporter), which may then affect program effectiveness [28, 36, 42].

Logistical challenges around providing support to healthcare workers in a hospital setting are difficult, especially in the constrained environment created by the COVID-19 pandemic. Logistical challenges were noted by coach participants in this data, elsewhere in our research [15], as well as in other program evaluations [38, 39, 41]. Despite the challenges, we believe mental health support for HCWs is vital, especially since the mental health consequences of past epidemics is known to have been substantial [16–22]. In considering recovery from this pandemic, Palmara and Sinsky outline key questions to help guide recovery efforts, that encourage reflection and forward movement for both staff and leaders. The questions are reflective of the past experiences of the pandemic, but also emphasize the importance of considering logistics, in asking staff to reflect on what prevents them from doing work they are proud of, and what can be done to help people move forward [43]. We believe logistical challenges related to providing mental health support in-house are better addressed at the leadership level than at the level of coaches or other support providers; leaders and managers can provide important pieces like protected time to access sessions, and dedicated locations for private meetings.

The Resilience Coach peer support meetings serve many purposes for the Resilience Coaches themselves. They provide an ‘anchor’ in the shared goal of staff mental health as described earlier, provide a forum to share strategies around logistics, and are also key in that they help coaches feel less alone and mitigate personal burnout. Another example of this sort of group in current literature is a ‘Community of Practice;’ Delgado et al. suggest group discussion in a peer support context about the challenges of hospital work is a key way to enhance moral resilience in staff, which has been threatened by the COVID-19 pandemic [23, 44, 45]. The peer support meetings are also important in this regard, as Resilience Coaching involves exposure to potentially traumatic or morally distressing situations where there is no clear resolution. Coaches face additional challenges such as holding onto intense affect and identifying with the same struggles facing their colleagues. All of this puts coaches or other support providers at increased risk for vicarious traumatization [46–50]; peer support is an important preventative tactic in this regard [13].

It is vital that staff support programs have processes for looking after their provider’s wellbeing and encourage their providers to do the same. In Resilience Coaching, coaches found that in addition to the peer support meetings, working in pairs enabled a shared experience that helped spread the logistical burden and facilitated a system of support. Other literature notes the importance of peer support for mental health providers, whether structured or ad hoc [13, 14, 51]. Another valuable approach to supporting providers may be having access to personal mentors who can debrief following difficult situations [13, 44].

Through this study, we highlight that mental health professionals delivering support during crises like the COVID-19 pandemic are themselves also experiencing the same issues as their colleagues, such as burnout, fear, and exhaustion [13, 14, 23, 24, 38, 40, 45]. Since the support they can provide frontline healthcare workers is very important, but they themselves are variably on the frontline, it is important leaders remember to also ensure that the work of support providers is governed by the same wellness recommendations (rotational schedules, presence of leadership, time and space to decompress) [1–3].

### Limitations

Resilience Coaching is a program rooted in the Department of Psychiatry at [hospital], and elements of this program may be contextually specific. As described, the Department of Psychiatry at this hospital has a long history of staff support [16–22], which allowed the development of this program to launch quickly and credibly. The history of consultation-liaison work within the hospital allowed many of the clinicians to leverage their relationships with clinical teams in a manner that felt organic and comfortable. Lastly, dedicated COVID-19 funding from the provincial agencies supported a more robust program than might otherwise have been possible in an exclusively fee for service model outside the support of an academic health sciences centre. Literature notes that all elements of staff support programs may not be easily transferrable to new contexts [29]; we recognize these specific elements stated here are particularly localized to this program. That being said, key learnings from this research are transferrable, such as the importance of relationships between supporters and those they support, and supporters with each other: the ‘anchored personalization’ approach described by DeBenigo and Kerrissey [28, 36].

The research team consisting of 5 coaches and 2 non-coaches. The presence of Resilience Coaches as research leaders is largely a strength, as it aligns methodologically with tenets of Resilience Coaching that prioritize relationships and connectedness. It allows for greater understanding of context and nuance in the research process; however, closeness of a researcher to the subject can allow some bias in the work. The presence of the health services [initials] and education [initials] researchers help mitigate this. The data analysis, therefore, contained elements of objectivity and embeddedness because of the composition of the team.

### Significance

This study is significant in that it provides insight into the experiences of implementing a staff support program at a multi-site academic hospital, through the perspective of the providers. The experiences of those providing support are currently underrepresented in research related to supporting staff during the COVID-19 pandemic [13, 14].

This and other research suggests hospitals implementing staff support programs should consider developing training sessions or documents to help improve confidence of providers, and allow time for regular peer support meetings between providers, or develop other mechanisms for them to feel connected to each other [13, 14, 24–27, 51]. Difficulties expressed by some Resilience Coaches in providing support when they themselves felt burnt out, which was echoed in other literature [13, 14, 40, 51], suggests a need for a large roster of providers, so coaches may take breaks, or work in paired contexts. Interestingly, these recommendations echo some of those developed for frontline HCWs in literature, further emphasizing that support providers are also HCWs working in distressing conditions, whose experiences merit attention [1–5, 13, 14, 26, 51].

## Conclusion

Resilience Coaching is a model for supporting psychological wellbeing amongst hospital staff during a pandemic that prioritizes relationships, internal embeddedness, and support for coaches. Further research is needed to determine the extent to which this relationship rooted model is translatable to other healthcare contexts with differing institutional histories, or even other sectors such as education.

## Data Availability

The datasets used and/or analysed during the current study are not publicly available because consent was not obtained from participants that would allow for public storage of the data. Data is available from the corresponding author on reasonable request.

## Footnotes

1 As a linguistic note, in reporting results in this paper, we refer to people who participated in the interviews as ‘coaches,’ in preference over distanced language such as participant or informant. This choice is intentional, to help emphasize that these narratives provide a unique provider perspective on support programs developed and delivered during COVID-19. It is important to note, however, that when we use the plural ‘coaches’, we are referring only to the subset of coaches who participated in interviews, and not the entire roster of active Resilience Coaches.

2 This participant was referencing a custom that took place in the region during the first 6 months of the pandemic, where non-HCWs were encouraged to bang pots and pans at 7:00 pm in the evening, to say thank you for the hard work of HCWs.
